# Ten-year cardiovascular risk in diabetes patients without obstructive coronary artery disease: a retrospective Western Denmark cohort study

**DOI:** 10.1186/s12933-021-01212-x

**Published:** 2021-01-21

**Authors:** Kevin Kris Warnakula Olesen, Morten Madsen, Christine Gyldenkerne, Pernille Gro Thrane, Troels Thim, Lisette Okkels Jensen, Hans Erik Bøtker, Henrik Toft Sørensen, Michael Maeng

**Affiliations:** 1 Department of Cardiology, Aarhus University Hospital, Palle Juul-Jensens Boulevard 99, 8200 Aarhus N, Denmark; 2 Department of Clinical Epidemiology, Aarhus University Hospital, Olof Palmes Allé 43-45, Aarhus N, 8200 Denmark; 3 Department of Cardiology, Viborg Regional Hospital, Heibergs Allé 4A, Viborg, 8800 Denmark; 4 Department of Cardiology, Odense University Hospital, J.B. Winsløws Vej 4, Odense, 5000 Denmark

**Keywords:** Coronary angiography, Coronary artery disease, Death, Diabetes, Ischemic stroke, Myocardial infarction

## Abstract

**Background:**

Diabetes patients without obstructive coronary artery disease as assessed by coronary angiography have a low risk of myocardial infarction, but their myocardial infarction risk may still be higher than the general population. We examined the 10-year risks of myocardial infarction, ischemic stroke, and death in diabetes patients without obstructive coronary artery disease according to coronary angiography, compared to risks in a matched general population cohort.

**Methods:**

We included all diabetes patients without obstructive coronary artery disease examined by coronary angiography from 2003 to 2016 in Western Denmark. Patients were matched by age and sex with a cohort from the Western Denmark general population without a previous myocardial infarction or coronary revascularization. Outcomes were myocardial infarction, ischemic stroke, and death. Ten-year cumulative incidences were computed. Adjusted hazard ratios (HR) then were computed using stratified Cox regression with the general population as reference.

**Results:**

We identified 5734 diabetes patients without obstructive coronary artery disease and 28,670 matched individuals from the general population. Median follow-up was 7 years. Diabetes patients without obstructive coronary artery disease had an almost similar 10-year risk of myocardial infarction (3.2% vs 2.9%, adjusted HR 0.93, 95% CI 0.72–1.20) compared to the general population, but had an increased risk of ischemic stroke (5.2% vs 2.2%, adjusted HR 1.87, 95% CI 1.47-2.38) and death (29.6% vs 17.8%, adjusted HR 1.24, 95% CI 1.13–1.36).

**Conclusions:**

Patients with diabetes and no obstructive coronary artery disease have a 10-year risk of myocardial infarction that is similar to that found in the general population. However, they still remain at increased risk of ischemic stroke and death.

## Introduction

Patients with diabetes are considered to have a greater risk of cardiovascular disease than individuals without diabetes [1]. Diabetes patients with coronary artery disease (CAD) also have a substantially higher risk of subsequent adverse cardiovascular outcomes compared to non-diabetes patients with CAD [2]. Still, earlier studies have found that diabetes and non-diabetes patients have the same low risk of myocardial infarction (MI), in the absence of obstructive CAD, as assessed by coronary angiography (CAG) or coronary computed tomography angiography [2–4]. However, prior studies compared symptomatic routine clinical care patients referred for coronary examination and were limited by lack of generalizability to the general population. The cardiovascular risk of diabetes patients without CAD compared to the general population remains largely unknown.

We hypothesized that diabetes patients without obstructive CAD have a risk of MI similar to that of the general population. To address this question, we conducted a cohort study in which we compared diabetes patients without CAD, as assessed by CAG, in Western Denmark to an age- and sex matched cohort without history of MI from the Western Denmark general population.

## Methods

### Databases

We conducted a retrospective, registry-based cohort study. Denmark has a free tax-supported health care system. This cohort study was based on data from several databases. The Western Denmark Heart Registry has recorded every CAG performed in Western Denmark since 1999 [5]. The Danish Civil Registration System contains information on the vital status of every individual residing in Denmark [6]. Each resident is assigned a personal unique 10-digit number at birth or upon immigration. This identifier is used in every registry in Denmark, allowing linkage of patient information among health care registries. The Danish National Patient Registry has recorded each Danish resident’s contacts with the public hospital system, including outpatient clinics, since 1977. This registry contains information on primary and secondary discharge diagnoses based on ICD-8 and ICD-10 codes [7]. The Danish Prescription Registry has registered every prescription redeemed at any Danish pharmacy since 1994 [8].

### CAG diabetes cohort

**Fig. 1:**
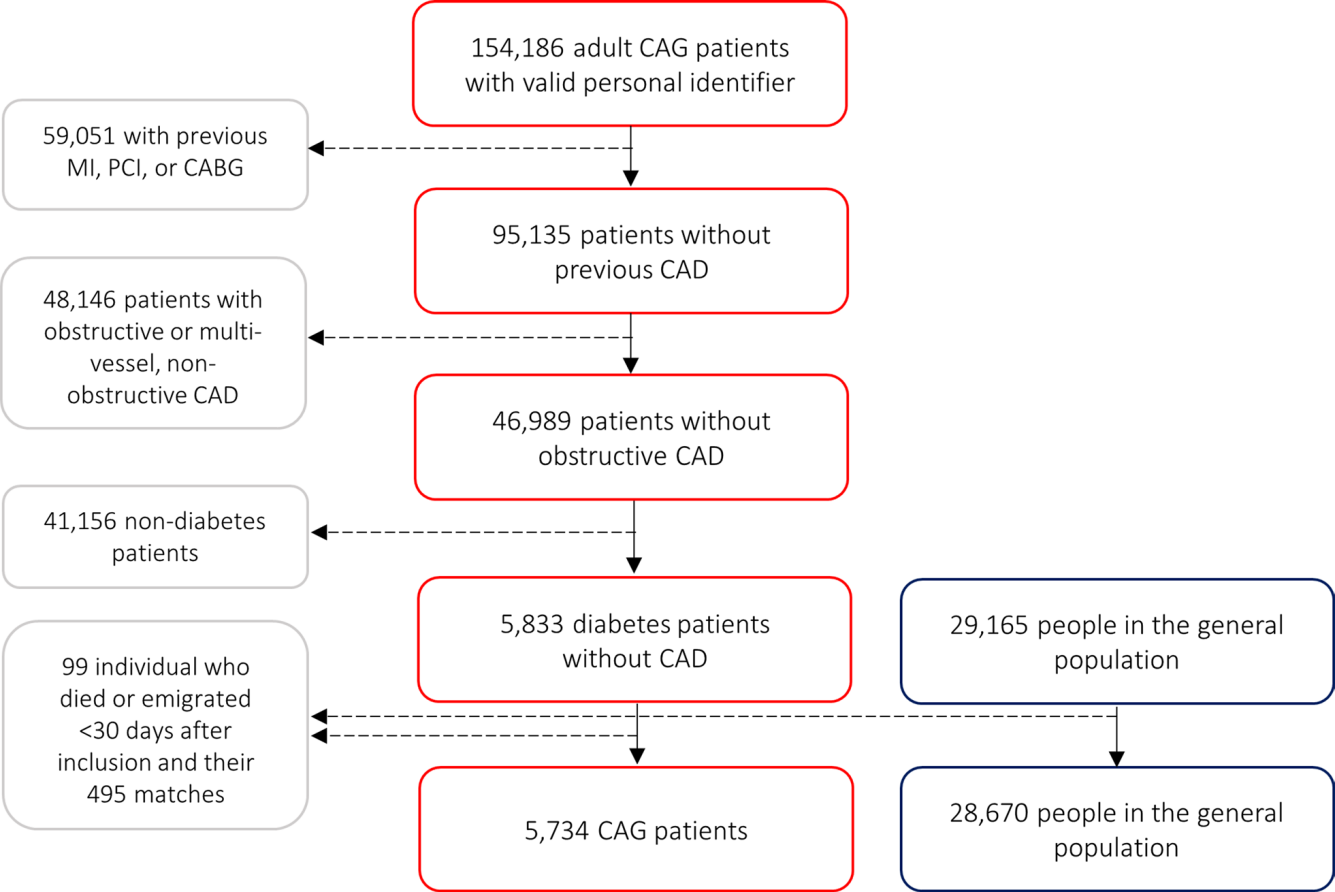
Patient selection

We reviewed every patient examined using CAG between January 1, 2003 and December 31, 2016 in Western Denmark (Fig. 1). If the same patient received multiple examinations, the first CAG was considered the index procedure. We excluded patients with a previous history of MI, percutaneous coronary intervention (PCI), and/or coronary artery bypass grafting (CABG) recorded in either the Western Denmark Heart Registry or the Danish National Patient Registry prior to CAG. Patients with obstructive CAD (≥ 50% coronary stenosis in ≥ 1 coronary vessel) or diffuse CAD (defined as non-obstructive CAD in ≥ 2 coronary vessels) also were excluded. Finally, only patients with either registered diabetes prior to inclusion in the Danish National Patient Registry or the Western Denmark Heart Registry, or in active anti-diabetes treatment [≥ 1 redeemed prescription(s) registered in the Danish Prescription Registry 6 months before or 30 days after CAG] were included (Additional file 1: Table S1).

### General population cohort

Diabetes patients were frequency matched by sex and age in a 1:5 ratio with individuals drawn from the Western Denmark general population who had no previous history of MI, PCI, or CABG in the Danish National Patient Registry at the time of matching. Replacement was allowed during matching [9].

### Co-medications

Active drug treatment was defined as redeeming ≥ 1 prescription(s) 6 months before or 30 days after study inclusion (Additional file 1: Table S1) [8]. Diabetes duration was estimated as the time from the first insulin or non-insulin prescription dispensation recorded in the Danish National Prescription Registry until start of follow-up [8]. Duration of diabetes as categorized as 0–4 years, 5–9 years, and  ≥ 10 years.

### Comorbidity

Disease burden prior to start of follow-up was ascertained from the Danish National Patient Registry using inpatient and outpatient primary and secondary discharge diagnoses (Additional file 1: Table S1). In the matched general population sample, diabetes was identified by combining discharge diagnoses and dispensed diabetes prescriptions.

### Outcomes

MI was defined as a primary or secondary discharge diagnosis during an acute hospitalization registered in the Danish National Patient Registry (Additional file 1: Table S1) [10]. Ischemic stroke was defined as a primary or secondary discharge diagnosis recorded in the Danish National Patient Registry [11]. Deaths were identified from the Civil Registration System [6].

### Statistical analysis

Follow-up started 30 days after study inclusion to reduce the risk of double-registration of CAG-related ischemic events. People with  < 30 days of follow-up, due either to death or emigration, were excluded from analyses, as well as their matched counterparts. Follow-up continued until an outcome, death, emigration, or end of follow-up (December 31, 2018). Follow-up was capped at 10.0 years, corresponding to the 75th percentile of overall follow-up. Events beyond 10 years were censored. Cumulative incidence proportion (CIP) curves were constructed. Ten-year CIP curves and risk differences (RDs) were computed, accounting for the competing risk of death in the case of MI and ischemic stroke. A stratified Cox regression analysis was used to compute the HR of death with 95% CI. The proportional hazards assumption was evaluated using log–log plots, which was satisfied [12]. Adjusted Cox regression analyses were performed, adjusting for statin treatment, antiplatelet treatment, oral anticoagulant treatment, and MI within 30 days after study inclusion (Model 1), and further adjusted for the following diabetes-related comorbidities: hypertension, peripheral artery disease, previous ischemic stroke, and chronic obstructive pulmonary disease (Model 2). For ischemic stroke and death, Model 2 also included heart failure, and atrial fibrillation. The general population was used as reference. Stata/MP 16 (StataCorp LLC, College Station, Texas, USA) was used for all analyses.

### Stratified analyses

Participants were stratified according to sex. We also stratified diabetes patients by type of diabetes treatment (non-pharmacological treatment, non-insulin treatment, and insulin treatment ± non-insulin treatment), and performed a stratified analysis by duration of diabetes (0–4 years, 5–9 years, ≥ 10 years).

### Subgroup analyses

A subgroup analysis was limited to diabetes patients referred for elective CAG with a procedural indication of stable angina pectoris, as well as to patients with an indication of acute coronary syndrome (ACS). We also performed a subgroup analysis in which we compared the diabetes cohort with individuals diagnosed with diabetes in the general population.

### Ethical considerations

This study was approved by the Danish Data Protection Agency (record no. 1-16-02-193-18). Observational, non-interventional, registry-based studies do not require approval from ethics committees or informed consent from patients according to Danish regulations.

## Results

A total of 5734 diabetes patients with no CAD as assessed by CAG were matched with 28,670 individuals from the Western Denmark general population (Fig. 1). Median follow-up time was 7.0 years (IQR 2.6–10.0 years).

### Baseline characteristics

Median age was 62 years (Table 1). Diabetes patients primarily underwent CAG due to stable angina pectoris, valve disease, or cardiomyopathy. Diabetes patients were more often diagnosed with chronic pulmonary disease, hypertension, heart failure, and atrial fibrillation compared to the general population group. We observed that 7.1% of matched persons from the general population had diabetes. The CAG cohort of diabetes patients also was more frequently treated with anti-thrombotic agents, oral anti-coagulants, statins, and anti-hypertensive drugs.

**Table 1:** Baseline characteristics of patients with diabetes and their matched general population comparison cohort

	Diabetes patients (n = 5734)		General population (n = 28,670)	
	N	%	N	%
Male sex	2997	52.3	14,985	52.3
Median age (IQR)	62 years (53-70)		62 years (53-70)	
Family history of ischemic heart disease^a^				
Yes	2058	35.9	–	–
Missing	670	11.7	–	–
Smoking^a^				
Active	1070	18.7	–	–
Former	2024	35.3	–	–
Never	2008	35.0	–	–
Missing	632	11.0	–	–
CAG procedural indication^a^				
STEMI	112	2.0	–	–
NSTEMI	259	4.5	–	–
Unstable AP	190	3.3	–	–
Stable AP	2666	46.5	–	–
Arrhythmia	228	4.0	–	–
Valvular disease	665	11.6	–	–
Cardiomyopathy	613	10.7	–	–
Other	899	15.7	–	–
Missing	102	1.8	–	–
Comorbidity				
Diabetes	5734	100.0	2041	7.1
Chronic pulmonary disease	626	10.9	1123	3.9
Hypertension	3362	58.6	3110	10.8
Peripheral artery disease	214	3.7	456	1.6
Heart failure	991	17.3	262	0.9
Atrial fibrillation	1070	18.7	979	3.4
Ischemic stroke	201	3.5	285	1.0
Hemorrhagic stroke	12	0.2	69	0.2
MI < 30 days after inclusion	2	0.0	6	0.0
Comedication				
Aspirin	3535	61.6	3455	12.1
ADP-inhibitors	261	4.6	286	1.0
Vitamin K-antagonists	909	15.9	830	2.9
DOAC	182	3.2	148	0.5
Statin	4140	72.2	5423	18.9
Beta-blocker	3118	54.4	3227	11.3
ACE-inhibitor	2659	46.4	3832	13.4
ARB	1698	29.6	2870	10.0
Thiazide	1209	21.1	2879	10.0
Calcium-blocker	2050	35.8	3669	12.8
Insulin	1631	28.4	568	2.0
Non-insulin anti-diabetes agents	3658	63.8	1484	5.2

*ACE* angiotensin converting enzyme, *ADP* adenosine diphosphate, *AP* angina pectoris, *ARB* angiotensin-II receptor blocker, *CAG* coronary angiography, *DOAC* direct oral anti-coagulant, *MI* myocardial infarction, *NSTEMI* non ST-elevation myocardial infarction, *SD* standard deviation, *STEMI* ST-elevation myocardial infarction

^a^Data provide by the Western Denmark Heart Registry. Unavailable for the general population

### Medicine changes

Aspirin treatment decreased by 1.1% after CAG compared to 6 months prior to the procedure (Table 2). However, this reflects that 13.0% of diabetes patients stopped redeeming aspirin prescriptions by 6 months post-CAG, while 11.9% of patients, who previously had not taken aspirin, initiated aspirin despite lack of obstructive CAD.

**Table 2:** Change in medical treatment from 6 months before to 6 months after coronary angiography in diabetes patients without coronary artery disease and with > 6 months of follow-up (n = 5661)

	Before^a^	After^b^	New users^c^	Former users^d^	Net change^e^ (±)
Aspirin	55.1%	53.9%	11.9%	13.0%	− 1.1%
Statins	66.6%	70.0%	9.4%	6.0%	+ 3.4%
Adenosine diphosphate-inhibitors	3.3%	4.6%	2.5%	1.2%	+ 1.3%
Vitamin-K antagonist	13.0%	19.3%	7.5%	1.2%	+ 6.3%
Direct oral anti-coagulants	2.3%	3.7%	1.7%	0.4%	+ 1.3%
Beta-blockers	46.5%	52.6%	13.0%	6.9%	+ 6.1%
Angiotensin converting enzyme-inhibitors	41.6%	43.5%	7.8%	5.9%	+ 1.9%
Angiotensin-II receptor blockers	28.4%	29.2%	4.2%	3.4%	+ 0.8%
Thiazide	19.4%	18.1%	4.5%	5.8%	− 1.3%
Calcium-blockers	32.0%	33.4%	7.7%	6.3%	+ 1.4%
Insulin	27.0%	29.9%	3.8%	0.9%	+ 2.9%
Non-insulin diabetes medication	61.6%	62.2%	4.6%	4.0%	+ 0.6%

^a^Before: redeemed ≥ 1 prescription within 6 months before angiography

^b^After: redeemed ≥ 1 prescription within 6 months after angiography

^c^New user: redeemed ≥ 1 prescription within 6 months after angiography, but not 6 months before

^d^Former user: redeemed ≥ 1 prescription within 6 months before angiography, but not 6 months after

^e^Net change: overall change in prescriptions from before to after angiography

### Myocardial infarction

**Fig. 2:**
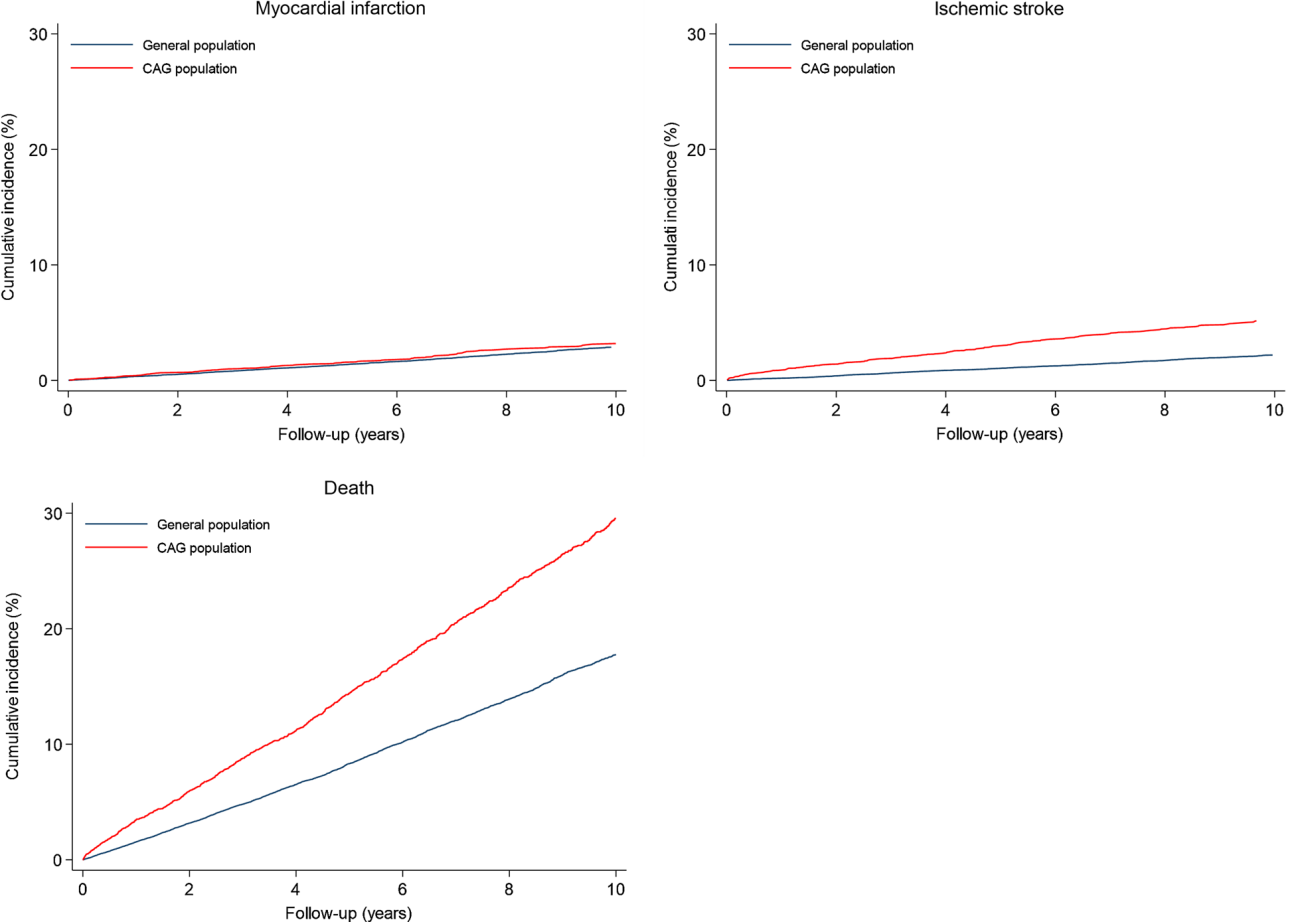
Ten-year cumulative incidence proportion of myocardial infarction, ischemic stroke, and death in patients with diabetes and a matched general population comparison cohort. The curves for myocardial infarction and ischemic stroke were adjusted for competing risk of death

**Fig. 3:**
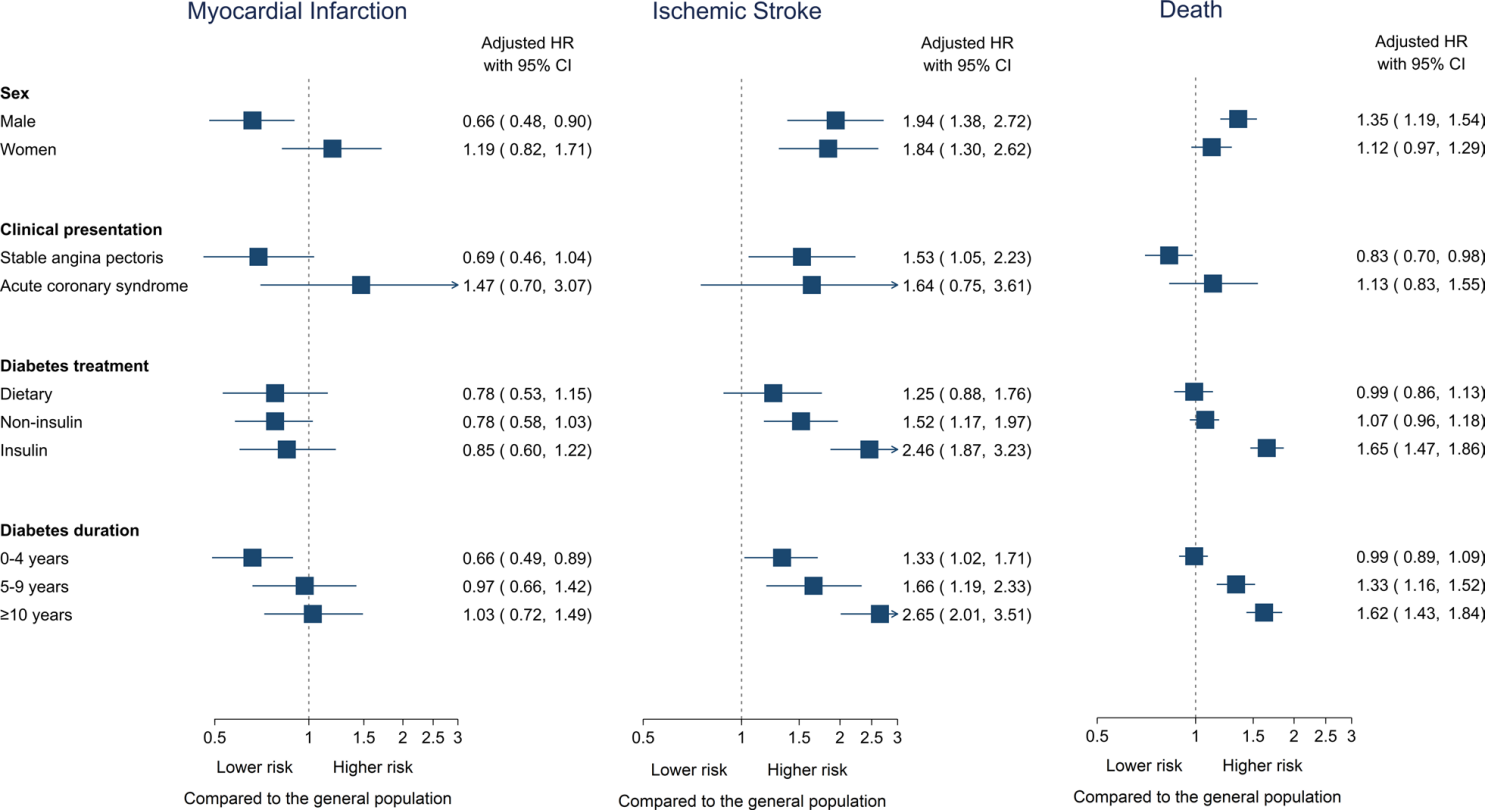
Stratified analysis by sex, clinical presentation, type of diabetes treatment, and diabetes duration. The hazard ratios (HR) denotes the risk as compared to a matched general population comparison cohort

Ten-year MI risks were low in both the diabetes cohort (3.2%) and the matched general population comparison cohort (2.9%) when accounting for death as a competing risk. Diabetes patients had a higher absolute 10-year MI incidence (RD 0.3%, 95% CI -0.3–0.9) (Table 3 and Fig. 2), but this small difference was no longer evident after adjusting for cardiovascular risk factors and prophylactic treatment (adjusted HR 0.93, 95% CI 0.72–1.20). When stratifying by sex, men with diabetes had a lower risk of MI than men in the general population cohort, while women had a similar MI risk as women in the general population cohort (Fig. 3 and Additional file 1: Table S2).

**Table 3:** Risk of myocardial infarction, ischemic stroke, and all-cause death in patients with diabetes and their matched general population comparison cohort

	Patients	Events	10-year cumulative incidence^a^ (95% CI)	Risk difference^a^ (95% CI)	Unadjusted HR (95% CI)	Adjusted HR^b^ (95% CI)	Adjusted HR^c^ (95% CI)
Myocardial infarction							
General population	28,670	595	2.9% (2.7–3.1)	Reference	Reference	Reference	Reference
Diabetes patients	5734	134	3.2% (2.7–3.8)	0.3% (-0.3–0.9)	1.26 (1.04–1.52)	1.07 (0.84–1.36)	0.93 (0.72–1.20)
Ischemic stroke							
General population	28,670	456	2.2% (2.0–2.4)	Reference	Reference	Reference	Reference
Diabetes patients	5734	230	5.2% (4.5–5.9)	3.0% (2.3–3.7)	2.96 (2.51–3.50)	2.04 (1.65–2.52)	1.87 (1.47–2.38)
Death							
General population	28,670	3659	17.8% (17.2–18.3)	Reference	Reference	Reference	Reference
Diabetes patients	5734	1228	29.6% (28.1–31.1)	11.8% (10.2–13.4)	1.88 (1.76–2.01)	1.58 (1.45–1.72)	1.24 (1.13–1.36)

*CAG* coronary angiography, *CI* confidence interval, *CIP* cumulative incidence proportion, *HR* hazard ratio

^a^Limited to the 75th percentile of follow-up (10 years). In myocardial infarction and ischemic stroke, accounting for the competing risk of death

^b^Adjusted for myocardial infarction within 30 days of angiography, statin treatment, oral anticoagulant treatment, and antiplatelet treatment

^c^Adjusted for peripheral artery disease, hypertension, chronic obstructive pulmonary disease, myocardial infarction within 30 days of angiography, statin treatment, oral anticoagulant treatment, and antiplatelet treatment. In case of ischemic stroke and death, additionally adjusted for congestive heart failure, previous ischemic stroke/TIA, and atrial fibrillation

### Ischemic stroke

Ten-year ischemic stroke incidence was higher in the diabetes cohort (5.2%) than in the matched general population cohort (2.2%) when accounting for death as a competing risk. This corresponded to a RD of 3.0% (95% CI 2.3–3.7), a difference that was sustained after adjustment for potential confounders.

### Death

Diabetes patients had higher mortality compared to the matched general population cohort (RD 11.8%, 95% 10.2–13.4). After adjusting for comorbidity and medical treatment, diabetes patients remained at increased risk of death compared to the matched general population cohort (adjusted HR 1.24, 95% CI 1.13–1.36).

### Subgroup analyses

When we restricted our analysis to diabetes patients with stable angina undergoing elective CAG, this subgroup had a low risk of both MI (adjusted HR 0.69, 95% CI 0.46–1.04) and death (adjusted HR 0.83, 95% CI 0.70–0.98) compared to their matched general population cohort. However, ischemic stroke risk remained elevated after adjustment (Fig. 3 and Additional file 1: Table S3).We also observed non-significant increases in risks of MI, ischemic stroke, and death in patients for whom ACS was the referral indication.

Type of diabetes treatment did not affect the risk of MI in the cohort of diabetes patients without obstructive CAD compared to the general population (Fig. 3 and Additional file 1: Table S4). Ischemic stroke risk and mortality were highest in diabetes patients treated with insulin and lowest in patients not receiving active pharmacological treatment compared to the general population (Fig. 3 and Additional file 1: Table S4). Duration of diabetes was associated with increased risk of MI, ischemic stroke, and death (Fig. 3 and Additional file 1: Table S5).

Compared to individuals from the general population with diabetes but no history of CAD, diabetes patients without CAD as assessed by CAG had a lower risk of MI and death and a similar risk of ischemic stroke (Additional file 1: Table S6).

## Discussion

Among diabetes patients without obstructive CAD, the 10-year risk of MI was only 0.3% higher than that observed in a general population comparison cohort, a difference which was no longer evident when the analysis was adjusted for comorbidity and use of prophylactic medications. Thus, a finding of no or mild CAD in diabetes patients undergoing CAG conferred an MI risk comparable to that for a general population comparison cohort. However, diabetes remained associated with increased risk of ischemic stroke and death despite absence of obstructive CAD and adjustment for comorbidity. Stratified analyses suggested that clinical presentation, diabetes treatment, and duration of diabetes were associated with relatively higher risks of ischemic stroke and death than observed in the matched general population cohort. Thus, while absence of CAD was associated with a low MI risk, diabetes patients had a higher risk of other cardiovascular outcomes, particularly in certain subgroups, despite more frequent treatment with preventive medications.

It has previously been shown that diabetes patients without obstructive CAD, as assessed by either CAG or coronary computed tomography angiography (CCTA), have similar MI risks as non-diabetes patients without CAD undergoing the same imaging procedures [2–4]. However, in earlier studies, the comparison group consisted of a selected group of symptomatic patients. It is unlikely that this group reflects the risk of MI in the general population without previous CAD. Our results therefore expand upon prior findings by showing that MI risk among diabetes patients is not only similar to that of non-diabetes patients undergoing CAG or CCTA, but also similar to that of a matched general population without known CAD. Of note, compared to individuals from the general population with diabetes, diabetes patients without CAD had a lower risk of both MI and death.

It is important to underscore that the risk of ischemic stroke was doubled in the diabetes cohort compared with the general population, despite lack of significant CAD. Moreover, the 10-year risk of ischemic stroke was higher (5%) than the risk of MI (3%). This is consistent with a recent report that also found that diabetes patients without angiographic CAD were at elevated risk of ischemic stroke [13]. The control group differed from this study, consisting of non-diabetes patients undergoing CAG [13]. These results may reflect thromboembolic events rather than manifestations of carotid and cerebral atherosclerotic disease, which again may be related to a higher prevalence of obesity in patients with diabetes [14].

Certain subgroups of diabetes patients were at increased cardiovascular risk despite lack of obstructive CAD. Referral for CAG due to suspected ACS, insulin treatment, and duration of diabetes were all associated with poor prognosis after the examination. Diabetes duration increases stroke risk by 3% per year [15]. While screening of asymptomatic high-risk diabetes patients is not currently recommended, asymptomatic patients with diabetes duration > 10.5 years may benefit from non-invasive angiographic screening [16, 17]. Insulin treatment has also been associated with increased stroke risk and higher mortality [13, 15, 18, 19]. These associations also apply to diabetes patients without obstructive CAD, as assessed by CAG, compared to the general population. Male diabetes patients without CAD had a lower relative risk of MI, while female patients had higher relative risk compared to same-sex members of the general population. However, this reflects sex-related difference in MI risk in the general population, where women (2.1%) are at a much lower risk than men (4.3%). The absolute risk was more similar among men and women after CAG (3.9% vs 3.6%) in the absence of CAD. Meta-analyses differ on whether sex affects cardiovascular risk in diabetes patients with established cardiovascular disease compared to non-diabetes patients [20, 21]. Among diabetes patients without obstructive CAD, we did not find clinical differences between sexes.

We observed a small net decrease of 1.1% in aspirin treatment after CAG, as 13% of all diabetes patients without CAD discontinued aspirin treatment and 12% initiated treatment after CAG. To some extent, this finding reflects initiation of aspirin during acute hospitalizations due to suspicion of acute coronary syndrome. It is common that aspirin treatment is continued following CAG. Still, more than half of diabetes patients without CAD were treated with aspirin after CAG. The relatively low prevalence of other vascular comorbidities [peripheral artery disease (3.7%) and ischemic stroke (3.5%)] cannot account for the continued high use of aspirin in these patients. With a 0.3% and 2.6% 10-year RD of MI and ischemic stroke compared to the general population, respectively, diabetes patients without obstructive CAD may not require continued antithrombotic treatment unless otherwise indicated. Recent randomized clinical trials have questioned the role of aspirin in primary cardiovascular prevention [22, 23]. The ASCEND (A Study of Cardiovascular Events iN Diabetes) trial randomized 15,480 diabetes patients without a previous history of cardiovascular disease to either aspirin or placebo [22]. While aspirin reduced a composite of serious vascular events (MI, ischemic stroke, transient ischemic attack, and cardiovascular death) by 1.1%, aspirin had no impact on MI risk (HR 0.98, 95% CI 0.80–1.19). The reduction in cardiovascular events was offset by a 0.9% increase in major bleeding. Further, a large meta-analysis of 23,488 diabetes patients found no effect of aspirin in primary prevention of MI (HR 0.94, 95% CI 0.83–1.07), while significantly increasing major bleeding risk (HR 1.29, 95% CI 1.11–1.5) [24]. The American Diabetes Association does not recommend primary prevention with aspirin, except in high-risk diabetes patients with an acceptably low bleeding risk [25]. European guidelines suggest a similar approach in recommending aspirin accompanied by prophylactic proton pump inhibition in ‘high’ and ‘very high’ risk diabetes patients without previous vascular disease [26]. The high rate of aspirin treatment in diabetes patients at documented low risk of MI may confer a bleeding risk rather than add protection against future ischemic events. Statin therapy is recommended to most patients with diabetes, and have in observational studies been found to reduce MI, cardiac death, and all-cause mortality in patients without obstructive CAD by CCTA [27–29]. Continued statin treatment should be considered in diabetes patients despite absence of obstructive CAD.

Patients registered with 0-vessel disease in the Western Denmark Heart Registry have either no CAD or non-obstructive CAD in a single vessel, but the registry does not differentiate between the two conditions. A large American cohort study found no difference in 1-year MI risk between diabetes patients with no apparent CAD versus diabetes patients with non-obstructive single vessel disease [30]. However, patient-level differences in non-obstructive plaque extent could potentially be used in additional risk stratification of diabetes patients without obstructive CAD [31]. CCTA can be used to identify diabetes patients with an incremental risk of cardiovascular disease based on CAD severity [3, 32]. Non-obstructive CAD is associated with coronary microvascular dysfunction, particularly in women with poor glycemic control, which significantly increases the risk of major adverse cardiovascular events [33–35]. Diabetes increases cardiovascular risk in patients with non-obstructive CAD and ACS, supporting our results in which diabetes patients referred to CAG with ACS had increased risk of cardiovascular outcomes compared to the general population [36, 37].

## Limitations

Our observational study provides data on the cardiovascular risk in Danish patients with diabetes compared to the Danish general population. The results, however, may not be representative for patients with diabetes or general population individuals in other countries with different health care systems and ethnicities. We did not have access to HbA1c measurements, which have been found to correlate with cardiovascular risk [38]. However, intensive glucose control has not been found uniformly to reduce cardiovascular risk [39–41]. Another concern is that our reference cohort consisted of a random sample of individuals from the Western Denmark general population without *known* coronary artery disease, but some may in fact have significant CAD. Hence, comparing angiographic absence of CAD with lack of diagnosed CAD reflects a clinical approach rather than an assumption of similar extent of CAD.

## Conclusions

In conclusion, diabetes patients without CAD as assessed by CAG had a similar risk of MI, but an increased risk of ischemic stroke and death, compared to a general population cohort. Medical treatment of diabetes, especially with insulin, and long duration of diabetes may exacerbate the risk of ischemic stroke and death compared to that experienced by a general population cohort. Despite absence of obstructive CAD and a low risk of MI, diabetes still remains associated with other cardiovascular outcomes.

## Supplementary information

**Additional file 1: Table S1.** International Classification of Diseases (ICD) from the Danish National Patient Registry [7]. **Table S2.** Risk of myocardial infarction, ischemic stroke, and all-cause death stratified by sex. **Table S3.** Risk of myocardial infarction, ischemic stroke, and all-cause death stratified in patients referred with stable angina pectoris and acute coronary syndrome. **Table S4.** Risk of myocardial infarction, ischemic stroke, and all-cause death in diabetes patients stratified by type of diabetes treatment and the general population. **Table S5.** Risk of myocardial infarction, ischemic stroke, and all-cause death in diabetes patients without coronary artery disease by duration of diabetes treatment**. Table S6.** Risk of myocardial infarction, ischemic stroke, and all-cause death compared to individuals from the general population with diabetes.

## Data Availability

According to Danish data protection regulations, data cannot be made publicly available.

## Abbreviations

ASCEND: A Study of Cardiovascular Events iN Diabetes
ACS: Acute coronary syndrome
CABG: Coronary artery bypass grafting
CAG: Coronary angiography
CAG: Coronary artery disease
CCTA: Coronary computed tomography angiography
CIP: Cumulative incidence proportion
MI: Myocardial infarction
PCI: Percutaneous coronary intervention
RD: Risk difference
