# Supplementary material for: Ten-year cardiovascular risk in diabetes patients without obstructive coronary artery disease: a retrospective Western Denmark cohort study
Source: Cardiovasc Diabetol. 2021 Jan 21;20:23. doi: 10.1186/s12933-021-01212-x (PMC7819163; doi:10.1186/s12933-021-01212-x)
Supplement: Supplementary file 1 — Additional file 1: Table S1. International Classification of Diseases (ICD) from the Danish National Patient Registry [7]. Table S2. Risk of myocardial infarction, ischemic stroke, and all-cause death stratified by sex. Table S3. Risk of myocardial infarction, ischemic stroke, and all-cause death stratified in patients referred with stable angina pectoris and acute coronary syndrome. Table S4. Risk of myocardial infarction, ischemic stroke, and all-cause death in diabetes patients stratified by type of diabetes treatment and the general population. Table S5. Risk of myocardial infarction, ischemic stroke, and all-cause death in diabetes patients without coronary artery disease by duration of diabetes treatment. Table S6. Risk of myocardial infarction, ischemic stroke, and all-cause death compared to individuals from the general population with diabetes. [file 12933_2021_1212_MOESM1_ESM.docx]

SUPPLEMENTAL MATERIAL

Ten-Year Cardiovascular Risk in Diabetes Patients without Obstructive Coronary Artery Disease – a Western Denmark Cohort Study

Kevin Kris Warnakula Olesen MDa,b (ORCID iD: 0000-0002-0560-3615), Morten Madsen MScb, Christine Gyldenkerne MDa, Pernille Gro Thrane MDa, Troels Thim MD PhDa, Lisette Okkels Jensen MD DMScic, Hans Erik Bøtker MD DMScia, Henrik Toft Sørensen MD DMScib, Michael Maeng MD PhDa

a) Department of Cardiology, Aarhus University Hospital, Aarhus, Denmark

b) Department of Clinical Epidemiology, Aarhus University Hospital, Aarhus, Denmark

c) Department of Cardiology, Odense University Hospital, Odense, Denmark

| **Table S1. International Classification of Diseases (ICD) from the Danish National Patient Registry** | | |
| --- | --- | --- |
| *Diagnosis* | *ICD-8 code* | *ICD-10 code* |
| Myocardial infarction |  | DI21* |
| Ischemic stroke |  | DI63*, DI64* |
| Atrial fibrillation |  | DI48* |
| Hypertension |  | DI10*, DI15.1, DI15.8, DI15.9 |
| Heart failure |  | DI11.0, DI13.0, DI13.2, DI50* |
| Peripheral artery disease including intermittents claudication |  | DI70, DI70.1-DI70.9. DI73.9* |
| Moderate to severe renal disease |  | DI12*, DI13*, DN00-DN05*, DN07*, DN11*, DN14*, DN17-DN19*, DQ61* |
| Diabetes |  | DE10*-DE14*, DH36.0, DO24.0-24.3, DO24.5-24.9 |
| PCI | 30350, 30354, 30359 | KFNG02*, KFNG05*, KFNG96 |
| CABG | 30009, 30019, 30029, 30039, 30049, 30059, 30069, 30079, 30089, 30099, 30109, 30119, 30120, 30129, 30139, 30149, 30159, 30169, 30179, 30189, 30199, 30200 | KFNA*-KFNE* |

| **Table S1 (cont.) Anatomical Therapeutic Chemical (ATC) Classification System codes from the Danish Prescription Registry** | |
| --- | --- |
| *Drug* | *ATC code* |
| Non-vitamin K oral anticoagulants | B01AF02, B01AE07, B01AF01, B01AF03 |
| Vitamin K-antagonists | B01AA03, B01AA04 |
| Aspirin | B01AC06, N02BA01 |
| ADP inhibitors | B01AC04, B01AC22, B01AC24, B01AC25 |
| Statin | C10AA* |
| β-blocker | C07* |
| Angiotensin converting enzyme inhibitors | C09A*, C09B* |
| Angiotensin-II receptor blockers | C09C*, C09D* |
| Thiazide | C03AA*, C03AB* |
| Calcium channel antagonist | C08C*, C08D* |
| Insulin | A10A* |
| Non-insulin diabetes medication | A10B* |

| **Table S2.** **Risk of myocardial infarction and all-cause death stratified by sex** | | | | | |  |  |
| --- | --- | --- | --- | --- | --- | --- | --- |
|  | Patients | Events | CIP^*^  (95% CI) | Risk difference^*^  (95% CI) | Unadjusted HR (95% CI) | Adjusted HR^†^ (95% CI) | Adjusted HR^‡^ (95% CI) |
| *Men* | | | | | | | |
| *Myocardial infarction* | |  |  |  |  |  |  |
| General population | 14,985 | 407 | 4.26% (3.84-4.72) | reference | reference | reference | reference |
| CAG population | 2,997 | 69 | 3.94% (3.03-5.10) | -0.32% (-1.43 – 0.79) | 0.97 (0.75–1.25) | 0.89 (0.65–1.23) | 0.66 (0.48–0.90) |
| *Ischemic stroke* | | | | | | | |
| General population | 14,985 | 252 | 2.63 (2.31-2.99) | reference | reference | reference | reference |
| CAG population | 2,997 | 131 | 6.28 (5.25-7.51) | 3.65% (2.48 – 4.81) | 3.03 (2.43–3.79) | 2.03 (1.52–2.71) | 1.94 (1.38–2.72) |
| *Death* |  |  |  |  |  |  |  |
| General population | 14,985 | 2,020 | 19.01% (18.22–19.84) | reference | reference | reference | reference |
| CAG population | 2,997 | 703 | 32.83% (30.67–35.09) | 13.83% (11.47–16.19) | 1.98 (1.81–2.16) | 1.71 (1.53-1.91) | 1.35 (1.19–1.54) |
| *Women* | | | | | | | |
| *Myocardial infarction* | | | | | | |  |
| General population | 13,685 | 211 | 2.06% (1.77-2.39) | reference | reference | reference | reference |
| CAG population | 2,737 | 39 | 3.61% (2.79-4.66) | 1.55% (0.58 – 2.52) | 1.85 (1.39–2.45) | 1.31 (0.91-1.89) | 1.19 (0.82-1.71) |
| *Ischemic stroke* | | | | | | | |
| General population | 13,685 | 204 | 2.26 (1.95-2.61) | reference | reference | reference | reference |
| CAG population | 2,737 | 99 | 5.52 (4.46-6.81) | 3.26 (2.03-4.48) | 2.87 (2.23–3.70) | 2.04 (1.49-2.79) | 1.84 (1.30-2.62) |
| *Death* |  |  |  |  |  |  |  |
| General population | 13,685 | 1,639 | 16.41% (15.65–17.21) | reference | reference | reference | reference |
| CAG population | 2,737 | 525 | 26.08% (24.05–28.24) | 9.64% (7.40–11.88) | 1.77 (1.59–1.96) | 1.44 (1.27-1.63) | 1.12 (0.97-1.29) |
| ^*^ Limited to 10 years of follow-up.  ^†^ Adjusted for statin treatment, oral-anticoagulant treatment, antiplatelet treatment.  ^‡^ Adjusted for previous ischemic stroke/TIA, peripheral artery disease, hypertension, chronic pulmonary disease, statin treatment, oral-anticoagulant treatment, antiplatelet treatment. Additionally adjusted for congestive heart failure and atrial fibrillation in the case ischemic stroke and death.  CAG: coronary angiography  CI: confidence interval  CIP: cumulative incidence proportion  HR: hazard ratio | | | | | | | |

| **Table S3.** **Risk of myocardial infarction and all-cause death stratified in patients referred with stable angina pectoris and acute coronary syndrome** | | | | | | | |
| --- | --- | --- | --- | --- | --- | --- | --- |
|  | Patients | Events | CIP^*^  (95% CI) | Risk difference^*^  (95% CI) | Unadjusted HR (95% CI) | Adjusted HR^†^ (95% CI) | Adjusted HR^‡^ (95% CI) |
| *Stable angina pectoris* | | | | | | | |
| *Myocardial infarction* | |  |  |  |  |  |  |
| General population | 12,310 | 275 | 2.91% (2.57-3.28) | reference | reference | reference | reference |
| CAG population | 2,462 | 57 | 2.97% (2.26-3.84) | 0.07% (-0.80 – 0.93) | 1.05 (0.78-1.40) | 0.78 (0.54-1.13) | 0.69 (0.46-1.04) |
| *Ischemic stroke* | | | | | | | |
| General population | 12,310 | 186 | 1.96% (1.68-2.26) | reference | reference | reference | reference |
| CAG population | 2,462 | 95 | 5.10% (4.12-6.21) | 3.14% (2.05 – 4.22) | 2.62 (2.03-3.37) | 1.52 (1.08-2.14) | 1.53 (1.05-2.23) |
| *All-cause death* |  |  |  |  |  |  |  |
| General population | 12,310 | 1,452 | 15.77% (15.00–16.58) | reference | reference | reference | reference |
| CAG population | 2,462 | 347 | 19.78% (17.88–21.85) | 4.01% (1.88–6.14) | 1.20 (1.07-1.36) | 0.97 (0.84-1.13) | 0.83 (0.70-0.98) |
| *Acute coronary syndrome* | | | | | | | |
| *Myocardial infarction* | | | | | | |  |
| General population | 2,805 | 56 | 2.50% (1.89-3.23) | reference | reference | reference | reference |
| CAG population | 561 | 18 | 4.37% (2.62-6.78) | 1.87% (-0.31 – 4.04) | 1.81 (1.05–3.13) | 1.58 (0.79-3.16) | 1.47 (0.70-3.07) |
| *Ischemic stroke* | | | | | | | |
| General population | 2,805 | 47 | 2.52% (1.85-3.36) | reference | reference | reference | reference |
| CAG population | 561 | 21 | 4.51% (2.84-6.75) | 1.99% (-0.09 – 4.07) | 2.75 (1.60-4.73) | 2.01 (1.03-3.93) | 1.64 (0.75-3.61) |
| *Death* |  |  |  |  |  |  |  |
| General population | 2,805 | 310 | 16.09% (14.40–17.95) | reference | reference | reference | reference |
| CAG population | 561 | 109 | 25.74% (21.62–30.50) | 9.65% (4.88–14.42) | 2.01 (1.60–2.53) | 1.47 (1.12-1.94) | 1.13 (0.83-1.55) |
| ^*^ Limited to the 75^th^ percentile of follow-up (10 years). Accounting for the competing risk of death.  ^†^ Adjusted for statin treatment, oral anticoagulant treatment, aspirin treatment, and myocardial infarction within 30 days after inclusion.  ^‡^ Adjusted for hypertension, previous ischemic stroke/TIA, peripheral artery disease, chronic pulmonary disease, statin treatment, oral anticoagulant treatment, aspirin treatment, and myocardial infarction within 30 days after inclusion. In case of ischemic stroke and death, further adjusted for heart failure and atrial fibrillation.  CAG: coronary angiography  CI: confidence interval  CIP: cumulative incidence proportion  HR: hazard ratio | | | | | | | |

| **Table S4.** **Risk of myocardial infarction, ischemic stroke, and all-cause death in diabetes patients stratified by type of diabetes treatment and the general population.** | | | | | | |
| --- | --- | --- | --- | --- | --- | --- |
|  | Patients | Events | CIP^*^  (95% CI) | Unadjusted HR (95% CI) | Adjusted HR^†^ (95% CI) | Adjusted HR^‡^ (95% CI) |
| *Myocardial infarction* | |  |  |  |  |  |
| General population | 28,670 | 595 | 2.89% (2.65-3.14) | reference | reference | reference |
| Dietary treatment | 1,209 | 30 | 3.09% (2.11-4.37) | 1.19 (0.83-1.72) | 0.92 (0.63-1.35) | 0.78 (0.53-1.15) |
| Non-insulin treatment | 2,894 | 64 | 3.24% (2.48-4.16) | 1.17 (0.91-1.52) | 0.90 (0.67-1.20) | 0.78 (0.58-1.06) |
| Insulin treatment | 1,631 | 40 | 3.33% (2.30-4.39) | 1.26 (0.91-1.73) | 1.02 (0.72-1.44) | 0.85 (0.60-1.22) |
| *Ischemic stroke* |  |  |  |  |  |  |
| General population | 28,670 | 456 | 2.19% (1.98-2.40) | reference | reference | reference |
| Dietary treatment | 1,209 | 40 | 3.85% (2.78-5.19) | 2.09 (1.51-2.89) | 1.37 (0.98-1.93) | 1.25 (0.88-1.76) |
| Non-insulin treatment | 2,894 | 102 | 4.73% (3.84-5.75) | 2.45 (1.98-3.04) | 1.54 (1.20-1.97) | 1.52 (1.17-1.97) |
| Insulin treatment | 1,631 | 88 | 6.88% (5.52-8.44) | 3.67 (2.92-4.61) | 2.63 (2.03-3.42) | 2.46 (1.87-3.23) |
| *Death* |  |  |  |  |  |  |
| General population | 28,670 | 3,659 | 17.75% (17.20-18.32) | reference | reference | reference |
| Dietary treatment | 1,209 | 249 | 25.71% (22.94-28.75) | 1.60 (1.41-1.82) | 1.26 (1.10-1.44) | 0.99 (0.86-1.13) |
| Non-insulin treatment | 2,894 | 554 | 29.71% (26.93-31.57) | 1.65 (1.51-1.80) | 1.32 (1.19-1.46) | 1.07 (0.96-1.18) |
| Insulin treatment | 1,631 | 425 | 33.77% (31.01-36.71) | 2.18 (1.97-2.41) | 2.10 (1.87-2.34) | 1.65 (1.47-1.86) |
| ^*^ Limited to the 75^th^ percentile of follow-up (10 years). Accounting for the competing risk of death.  ^†^ Model 1: Adjusted for age category, sex, statin treatment, oral anticoagulant treatment, aspirin treatment, and myocardial infarction within 30 days after inclusion.  ^‡^ Model 2: Adjusted for age category, sex, examination year, hypertension, previous ischemic stroke/TIA, peripheral artery disease, chronic pulmonary disease statin treatment, oral anticoagulant treatment, aspirin treatment, and myocardial infarction within 30 days after inclusion. In case of ischemic stroke and death, further adjusted for heart failure and atrial fibrillation.  CAG: coronary angiography  CI: confidence interval  CIP: cumulative incidence proportion  HR: hazard ratio | | | | | | |

| **Table S5. Risk of myocardial infarction, ischemic stroke, and all-cause death in diabetes patients without coronary artery disease by duration of diabetes treatment** | | | | | | | |
| --- | --- | --- | --- | --- | --- | --- | --- |
|  | Patients | Events | CIP^*^  (95% CI) | Unadjusted HR (95% CI) | Adjusted HR^†^ (95% CI) | Adjusted HR^‡^ (95% CI) |  |
| *Myocardial infarction* | |  |  |  |  |  |  |
| General population | 28,670 | 595 | 2.87% (2.65-3.14) | reference | reference | reference |  |
| 0-4 years | 3,170 | 64 | 2.70% (2.08-3.44) | 0.98 (0.76-1.27) | 0.77 (0.58-1.03) | 0.66 (0.49-0.89) |  |
| 5-9 years | 1,231 | 33 | 3.68% (2.52-5.15) | 1.46 (1.03-2.07) | 1.13 (0.78-1.64) | 0.97 (0.66-1.42) |  |
| >10 years | 1,333 | 37 | 4.06% (2.80-5.66) | 1.57 (1.13-2.19) | 1.22 (0.85-1.74) | 1.03 (0.72-1.49) |  |
| *Ischemic stroke* | |  |  |  |  |  |  |
| General population | 28,670 | 468 | 2.20% (1.99-2.42) | reference | reference | reference |  |
| 0-4 years | 3,170 | 106 | 4.03% (3.29-4.88) | 2.09 (1.69-2.59) | 1.38 (1.08-1.75) | 1.32 (1.02-1.71) |  |
| 5-9 years | 1,231 | 47 | 5.15% (3.79-6.80) | 2.73 (2.02-3.68) | 1.74 (1.26-2.41) | 1.66 (1.19-2.33) |  |
| >10 years | 1,333 | 79 | 8.31% (6.51-10.37) | 4.44 (3.50-5.64) | 2.83 (2.17-3.70) | 2.65 (2.01-3.51) |  |
| *Death* | | | | | | | |
| General population | 28,670 | 3,659 | 17.75% (17.20-18.32) | reference | reference | reference |  |
| 0-4 years | 3,170 | 604 | 25.49% (23.64-27.45) | 1.50 (1.38-1.64) | 1.26 (1.14-1.39) | 0.99 (0.89-1.09) |  |
| 5-9 years | 1,231 | 285 | 34.27% (30.81-37.99) | 2.05 (1.81-2.31) | 1.70 (1.49-1.93) | 1.33 (1.16-1.52) |  |
| >10 years | 1,333 | 339 | 36.05% (32.64-39.69) | 2.35 (2.10-2.62) | 1.92 (1.71-2.17) | 1.62 (1.43-1.84) |  |
| ^*^ Limited to the 75^th^ percentile of follow-up (10 years). In myocardial infarction and ischemic stroke, adjusted for the competing risk of death.  ^†^ Adjusted for age category, sex, examination year, statin treatment, oral anticoagulant treatment, and antiplatelet treatment.  ^‡^Adjusted for age category, sex, examination year, previous ischemic stroke/TIA, peripheral artery disease, hypertension, chronic pulmonary disease, statin treatment, oral anticoagulant treatment, and antiplatelet treatment. In case of ischemic stroke and death, additionally adjusted for congestive heart failure, and atrial fibrillation.  CAG: coronary angiography  CI: confidence interval  CIP: cumulative incidence proportion  HR: incidence rate ratio | | | | | | | |

| **Table S6.** **Risk of myocardial infarction, ischemic stroke, and all-cause death compared to individuals from the general population with diabetes.** | | | | | | | | | | |
| --- | --- | --- | --- | --- | --- | --- | --- | --- | --- | --- |
|  | Patients | Events | CIP^*^  (95% CI) | Unadjusted HR (95% CI) | Adjusted HR^†^ (95% CI) | | Adjusted HR^‡^ (95% CI) | | |  |
| *Myocardial infarction* | |  |  |  |  | |  | | |  |
| General population with diabetes | 2,041 | 61 | 5.07% (3.77-6.63) | reference | reference | | reference | | |  |
| Diabetes patients | 5,734 | 135 | 3.19% (2.66-3.79) | 0.68 (0.50–0.92) | 0.66 (0.47–0.91) | | 0.63 (0.45–0.88) | | |  |
| *Ischemic stroke* |  |  |  |  | |  | |  |  | |
| General population with diabetes | 2,041 | 69 | 4.85% (3.71-6.21) | reference | reference | | reference | | |  |
| Diabetes patients | 5,734 | 230 | 5.16% (4.51-5.88) | 1.07 (0.82–1.40) | 1.07 (0.80–1.43) | | 1.03 (0.77–1.39) | | |  |
| *Death* |  |  |  |  |  | |  | | |  |
| General population with diabetes | 2,041 | 463 | 34.82% (31.47–37.57) | reference | reference | | reference | | |  |
| Diabetes patients | 5,734 | 1,228 | 29.55% (28.06–31.11) | 0.83 (0.74–0.92) | 0.96 (0.86–1.08) | | 0.82 (0.51–0.92) | | |  |
| ^*^ Limited to the 75^th^ percentile of follow-up (10 years). In myocardial infarction and ischemic stroke, adjusted for the competing risk of death.  ^†^ Adjusted for statin treatment, oral anticoagulant treatment, and antiplatelet treatment.  ^‡^Adjusted for peripheral artery disease, hypertension, previous ischemic stroke/TIA, statin treatment, oral anticoagulant treatment, and antiplatelet treatment. In case of ischemic stroke and death, additionally adjusted for congestive heart failure, and atrial fibrillation.  CAG: coronary angiography  CI: confidence interval  CIP: cumulative incidence proportion  HR: hazard ratio | | | | | | | | | | |
